# Biological control interventions reduce pest abundance and crop damage while maintaining natural enemies in sub-Saharan Africa: a meta-analysis

**DOI:** 10.1098/rspb.2022.1695

**Published:** 2022-12-07

**Authors:** Fabrizia Ratto, Toby Bruce, Gilson Chipabika, Sithembile Mwamakamba, Rachel Mkandawire, Zeyaur Khan, Angela Mkindi, Jimmy Pittchar, Susannah M. Sallu, Stephen Whitfield, Kenneth Wilson, Steven M. Sait

**Affiliations:** ^1^ School of Biology, Faculty of Biological Sciences, University of Leeds, Miall Building, Leeds LS2 9JT, UK; ^2^ Sustainability Research Institute, School of Earth and Environment, University of Leeds, Miall Building, Leeds LS2 9JT, UK; ^3^ Department of Health Studies and Centre for Ecology, Evolution and Behaviour, School of Life Sciences and the Environment, Royal Holloway, University of London, Egham, Surrey TW20 0EX, UK; ^4^ School of Life Sciences, Keele University, Keele ST5 5BG, UK; ^5^ Zambia Agriculture Research Institute, Mulungushi House, Independence Avenue, Lusaka 10101, Zambia; ^6^ Food, Agriculture and Natural Resources Policy Analysis Network (FANRPAN), 141 Cresswell St Weaving Park, Pretoria, South Africa; ^7^ International Centre of Insect Physiology and Ecology, PO Box 30772-00100, Nairobi, Kenya; ^8^ School of Life Sciences and Bio-engineering, Department of Sustainable Agriculture, Biodiversity and Ecosystem Management, The Nelson Mandela African Institution of Science and Technology, PO Box 447- Arusha, Tanzania; ^9^ Lancaster Environment Centre, Lancaster University, Lancaster LA1 4YQ, UK

**Keywords:** conservation agriculture, predators, parasitoids, synthetic pesticides, botanical pesticides, insect pests

## Abstract

Insect pests are a major challenge to smallholder crop production in sub-Saharan Africa (SSA), where access to synthetic pesticides, which are linked to environmental and health risks, is often limited. Biological control interventions could offer a sustainable solution, yet an understanding of their effectiveness is lacking. We used a meta-analysis approach to investigate the effectiveness of commonly used biocontrol interventions and botanical pesticides on pest abundance (PA), crop damage (CD), crop yield (Y) and natural enemy abundance (NEA) when compared with controls with no biocontrol and with synthetic pesticides. We also evaluated whether the magnitude of biocontrol effectiveness was affected by type of biocontrol intervention, crop type, pest taxon, farm type and landscape configuration. Overall, from 99 studies on 31 crops, we found that compared to no biocontrol, biocontrol interventions reduced PA by 63%, CD by over 50% and increased Y by over 60%. Compared to synthetic pesticides, biocontrol resulted in comparable PA and Y, while NEA was 43% greater. Our results also highlighted that the potential for biocontrol to be modulated by landscape configuration is a critical knowledge gap in SSA. We show that biocontrol represents an effective tool for smallholder farmers, which can maintain yields without associated negative pesticide effects. Furthermore, the evidence presented here advocates strongly for including biocontrol practices in national and regional agricultural policies.

## 1. Introduction

One of the greatest global challenges of the twenty-first century is meeting the increasing demands for food production while minimizing adverse impacts on biodiversity and ecosystem health [1]. This challenge is particularly critical in sub-Saharan Africa (SSA) where the population is predicted to double over the coming decades [2], and food production is hampered by climate change impacts [3], which exacerbates significant yield losses already caused by crop pests [4,5]. For example, the invasion of the fall armyworm (*Spodoptera frugiperda*), which has caused crop losses of about $3 billion a year in SSA, has become one of the most important threats to maize production [6]. The fall armyworm is also a cause of major damage to other crops, including rice, sorghum, millet, cabbage and tomatoes, demonstrating the vulnerability of smallholder farming to crop pests.

Conventional synthetic pesticides have severe limitations as a means of pest control in SSA because they are economically inaccessible for a large portion of smallholder farmers in the region [7]. Pesticide residues also put human and livestock populations at risk from contaminated food and forage [8,9]. Furthermore, synthetic pesticides may lead to resistance in pest populations [10], and have negative impacts on non-target organisms, such as pollinators and natural enemies, and the ecosystem services that biodiversity provides in the production of food [11–13]. If the reduction of natural enemy populations is greater than that of the pest, this may lead to the resurgence of pests following pesticide applications [14], which is a widely reported problem associated with synthetic pesticides [15].

Biological control methods (hereafter biocontrol), which employ natural enemies of crop pests, have been adopted globally as an alternative approach to synthetic chemical pest control, and are often used as part of an integrated pest management strategy [16,17]. Extensive evidence is available on the responses of natural enemies to the landscape configuration surrounding crop fields [18], which reveals that landscape effects, albeit giving inconsistent responses, may be a key driver of pest regulation by natural enemies. Recent syntheses show consistent positive responses of natural enemies to landscape complexity [13], with higher natural enemy populations in complex versus simple landscapes [19] and a reduction of natural pest control in simplified landscapes [20].

However, meta-analyses of this kind are strongly biased in favour of the Northern Hemisphere, or they are global in scope, and so lack the scale of analysis that might be useful to policy makers in the SSA region. Furthermore, inputs such as chemicals fertilizers and pesticides are typically much less in Africa, and we would expect the effectiveness of biocontrol strategies to be different. There is a recognized need to develop evidence-based, environmentally friendly biocontrol management strategies in SSA, which boost capacities for their implementation across farming systems, locations and scales. This is exemplified by the Food and Agriculture Organization of the United Nations, who recognize that coordination and collaboration on fall armyworm control will require the implementation of environmentally sustainable pest management practices and policies at the regional, national and farmer-level [21].

In SSA, in addition to conventional biological control approaches that use live natural enemies such as predators, parasitoids and pathogens, smallholder farmers have recently adopted conservation biocontrol methods and plant-based botanical pesticides for the control of crop pests [22]. Conservation biocontrol methods include intercropping, push–pull technology and the maintenance of plant-rich field margins. Growing evidence highlights the potential of biocontrol interventions to reduce pest incidence and increase yield [23,24]. For example, push–pull technology has been shown to be effective against a range of crop pests, particularly maize stemborers [25] and botanical pesticides can reduce pest incidence and enhance yield in vegetable crops [26,27].

Although biocontrol interventions and botanical pesticides may provide sustainable and accessible alternatives to synthetic pesticides, their adoption by smallholder farmers has not been widespread [28]. This may be owing to knowledge gaps relating to their effectiveness and the factors that lead to their success or failure, particularly in comparison to synthetic pesticides. Biocontrol techniques have been applied to numerous crops and targeted a wide variety of pests in the region, yet there is a lack of understanding of how the effectiveness of biocontrol varies across different crop types and pest taxa [28]. Recent research in Tanzania found greater natural enemy diversity in fields surrounded by intercropped fields, suggesting spatial flow of potential biocontrol services across landscapes [29]. However, the established relationship between landscape configuration, natural enemies and pest regulation is almost entirely based on studies carried out in the global north and some global south regions [30], but very seldom in sub-Saharan regions where farmers are most exposed to food insecurity caused by crop pests [31]. More clarity is needed about the environmental factors affecting biocontrol and botanical pesticide performance in SSA to better assist in smallholder farmer decision-making, and to determine the broader indirect impact of pest management options on biodiversity compared to synthetic pesticides, both on a farm and at a landscape scale.

Quantitative analyses have been conducted on the performance of biocontrol agents [32], on the impact of landscape context on augmentative biocontrol [33] and pest and natural enemy responses [13]. However, none of these approaches have focussed specifically on the sub-Saharan region showing a geographical bias, nor have they evaluated the efficacy of different biocontrol interventions on pest populations and their damage to crops.

Here, we aim to better understand the key factors driving the success or failure of biocontrol interventions using quantitative meta-analysis. We broaden the definition of biocontrol interventions to encompass biological control using live organisms, as well as conservation agriculture and plant-derived botanical pesticides, which represent more recent pest control innovations. There has been very little assessment of their efficacy, especially botanical pesticides, as alternatives to synthetic chemical pesticides. Specifically, we posed the following questions: (i) what are the effects of biocontrol interventions on the management of insect crop pests in SSA? (ii) are these effects consistent across biocontrol techniques, crop types, target pests and farming systems? (iii) how does the effectiveness and impact of biocontrol interventions on crop pests and non-target insects compare to synthetic pesticides? and (iv) does the surrounding landscape configuration affect the efficacy of biocontrol interventions?

We hypothesized that pest abundance (PA) and crop damage (CD) would decrease, and crop yield (Y) would increase in crops subject to biocontrol interventions, that the impact on natural enemy abundance (NEA) would be less than that of synthetic chemical pesticides, and that these effects would be enhanced in fields surrounded by greater landscape complexity.

## 2. Material and methods

### (a) Data collection and inclusion criteria

To identify candidate studies, we screened a dataset included in a systematic map review carried out by Ratto *et al*. [28] that described the existing literature on biocontrol interventions for insect pests of crops in SSA. Ratto *et al.* [28] systematically searched Web of Science All Databases and Scopus, using a combination of search terms relating to a wide range of biocontrol techniques and insect pests (e.g. biocontrol, intercrop*, armyworm), agricultural settings (e.g. agri*, farm*) and the target geographical location (e.g. SSA, Southern Africa) (electronic supplementary material, table S1). The grey literature was captured by conducting additional searches on Google and Google Scholar and by searching websites of relevant institutions (electronic supplementary material, table S2). This mapping review covered a period between 2005 and April 2021 and was summarized narratively, with no quantitative analysis performed.

We integrated this initial dataset (149 articles) [28] with a follow up search of relevant papers published between April 2021 and December 2021 using the same search term combination. This search yielded 146 articles potentially appropriate for our review. We used the RepOrting standards for Systematic Evidence Syntheses (ROSES) [34] (electronic supplementary material, figure S1). Only articles published after 2005 were included to reflect modern biocontrol practices and to determine biocontrol effectiveness within a short timeframe. We focused on the sub-Saharan region, which has a large population of smallholder farmers who depend on local food production, and who suffer substantial incidences of insect pest outbreaks and CD that threatens their food security.

We included in the definition of biocontrol interventions any practice that uses natural enemies of pests, or chemical products derived from nature, for the control of pest populations. These include the augmentation, introduction or inoculation of natural enemies (i.e. predators, parasitoids and entomopathogens, such as bacteria, viruses and fungi), and conservation biocontrol (table 1). Conservation biocontrol was defined as the manipulation of habitat to enhance NEA and diversity [24] and included push–pull technology, intercropping and the maintenance of field margins. Botanical pesticides, defined as substances derived from natural materials (e.g. plant extracts), were also included.

**Table 1.** Definitions of biological control interventions included in the meta-analysis.

biocontrol intervention	description
botanical pesticides	insecticidal compounds in the form of water, oil or powder extracted from the leaves, seeds, pods, roots, bark, flower or fruits, of plants known to have pesticidal properties either from cultural knowledge or laboratory experiment
augmentation/introduction	increase the number of parasitoids, predators or entomopathogens by releasing the natural enemy (introduction, inoculation and inundation) or by supplying their food resources
intercropping	simultaneous cultivation of plant species in the same field for most of their growing period, e.g. cereal and beans or other food plants
push–pull	intercropping of maize or other crops with perennial fodder legumes (e.g. *Desmodium* spp.) to repel (push) pests. A trap crop, a perennial fodder (Napier grass or *Brachiaria* spp*.*) is planted around the plot to attract (pull) pests away from the crop
field margins	strip of land between the crop and the field boundaries sown with wildflowers and/or legumes, grass only or naturally regenerated
landscape effect	the effect of distance of cultivated areas to natural habitat, non-crop habitat and/or landscape complexity on the delivery of biocontrol

To ensure biologically meaningful comparisons, we applied further inclusion criteria. Only articles that quantitatively measured biocontrol performance on the outcome measures were included in the analysis. Only studies with replicated treatments at one or more sites were included. We screened studies wherein PA, CD, Y or NEA (hereafter ‘outcome measures’) were compared between crops following the implementation of a biocontrol intervention and untreated crops. We also extracted, where available, data on the outcome measures in crops treated with synthetic pesticides. Measures of CD included dead hearts (i.e. drying of the central shoot), damage to stems (e.g. stem tunnelling), pods, leaves, fruits, shoots that were specific to the target pests. Y was reported as either kg ha^−1^ or tonne ha^−1^, which was standardized to the latter for analysis.

We categorized the sites that had been exposed to a biocontrol intervention as ‘treatment’, with those that were left untreated as ‘negative control (−)’ and those treated with synthetic pesticides as ‘positive control (+)’. The mean, standard deviation and sample size of outcome measures were recorded for both the treatment and controls. When data were presented only in figures, we extracted data using ImageJ software [35]. We contacted the lead authors of the studies that had incomplete data.

**Figure 1.**
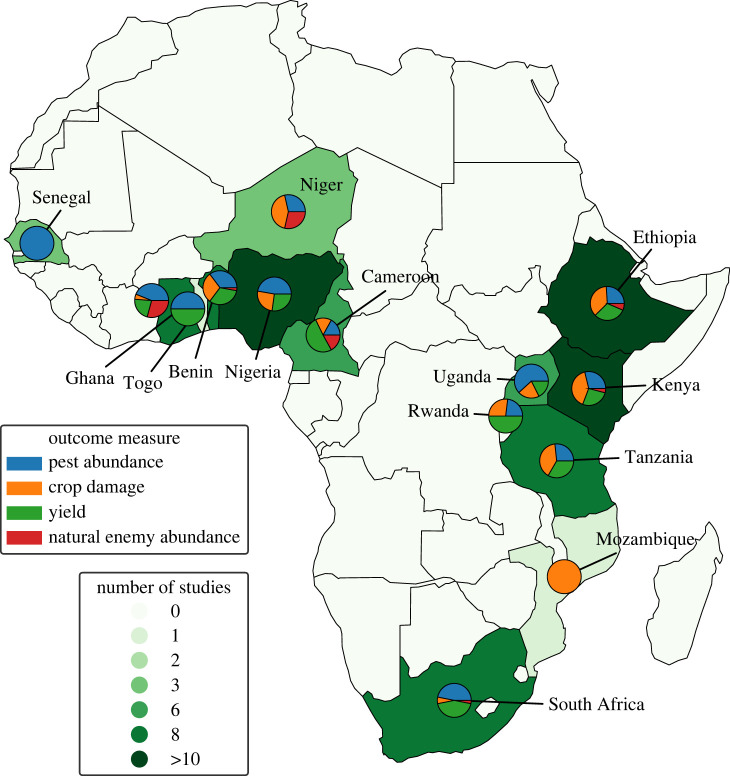
Geographical distribution map of studies included in the meta-analysis; colour coded by number of studies recorded per country. The pie charts show the outcome measures for each country, with blue, orange, green and red in the pie charts showing the proportion of outcomes for pest abundance, crop damage, yield and natural enemy abundance, respectively. (Online version in colour.)

For articles that presented multiple years of data sampling at the same site, we used the most recent data to control for non-independence of temporal data [36]. When the study was conducted in two or more spatially independent sites, we recorded them as independent observations. When a study presented outcome measures for several successive weeks, we averaged the means and recorded it as a single effect size. When different concentrations or different types of biocontrol agent were applied (e.g. entomopathogens and botanical pesticides), we used the highest concentration and recorded each biocontrol type as an independent observation. The screening resulted in a total of 99 articles and 512 studies included in the analysis (figure 1; electronic supplementary material, table S3 and figure S1).

### (b) Statistical analysis

In our meta-analysis, the log of the response ratio (ln*RR*) represents the influence of biocontrol interventions on the outcome measures and expresses the proportional difference between the treatment and the control groups [37]:

$$\ln RR=\ln(x_{1})\text{–}\ln(x_{2}),$$

where *x*_1_ is the mean of the outcome measure when biocontrol is applied (treatment), and *x*_2_ is the mean of the outcome measures under the untreated condition (control −) or after synthetic pesticide application (control +).

All outcome measures were analysed separately (PA, CD, Y and NEA). Fitted random effects models were used to calculate the overall means and 95% confidence intervals (CI) for each outcome measure to determine if biocontrol interventions significantly affected the outcome measures when compared to control areas (both untreated and pesticide treated). Random effect models do not assume that any variation in the effect size is due only to sampling error, and, instead, allow for a real random component of variation in effect size between studies (e.g. regional differences in study location). An effect of biocontrol intervention was considered significant if the 95% biased-corrected bootstrap CI of the effect size did not overlap zero [38].

Meta-regression was used to explore sources of heterogeneity across each dataset. Our analysis focussed on the following ecological, environmental and experimental parameters: (i) biocontrol technique; (ii) crop type; (iii) target pest taxon; and (iv) farming system. However, we could not use landscape complexity as a moderator as we found too few studies that investigated landscape context. To elucidate the variability of biocontrol efficacy across biocontrol techniques, we grouped studies according to whether they applied botanical pesticides, intercropping, field margins (border planting including legumes, sorghum or wild grasses), push–pull or augmentation/introduction methods. To determine if the effectiveness of biocontrol was dependent on crop type, we classified the study focus crops into cereal, fibre, fruits, vegetables and pulses. We did not include stimulants (e.g. coffee and cocoa) and nuts owing to small sample sizes. To establish whether biocontrol effectiveness varied across different pest insect taxa, we classified studies according to taxon of the targeted pest (Coleoptera, Hemiptera, Lepidoptera and Blattodea). Lastly, we classified studies into two field types: small farm (real smallholder farming conditions) and research farm (experimental field within a research centre), to identify any difference between these systems. Large commercial horticulture farms were not included in the meta-analysis as we primarily focussed on smallholder farmers and their food security. The above parameters were tested one by one as a sole moderator (i.e. fixed effects) for each outcome measure. To account for multiple comparisons from the same article, each model included ‘study’ nested within ‘article’ as random effects. The mean log response ratios and upper and lower bounds of 95% CI around the mean were back-transformed with the formula (e^lnR^-1) *100 and expressed as per cent change relative to the controls to facilitate interpretation.

We assessed publication bias in a number ways. We first visually assessed funnel plots for strong asymmetries (electronic supplementary material, figure S2) and ran Egger's regression test [39,40] and the trim-and-fill test [41]. Visual inspection of the funnel plots revealed symmetrical distribution of effect size around the meta-analytical mean of all outcome measures apart from PA. Egger's test indicated that publication bias was significant for the PA (*z* = −2.1065, *p* = 0.0352), which was inconsistent with the trim-and-fill tests that showed no missing studies for all datasets. Furthermore, we evaluated the sensitivity of our analysis by computing an influential case diagnostic and comparing fitted models with and without influential effect sizes; influential outliers were defined as those effect sizes whose hat values were two times larger than the average hat value and standardized residual values exceeding 3.0 [42] (electronic supplementary material, figures S3–S4). We also estimated the Rosenberg fail-safe number on all datasets, which is the number of non-significant unpublished studies required to eliminate a significant overall effect size (Rosenberg [43]). All statistical analyses were performed using the ‘metafor’ package in R (v. 4.1.2) [44].

## 3. Results

### (a) Comparison with no pest control

**Figure 2.**
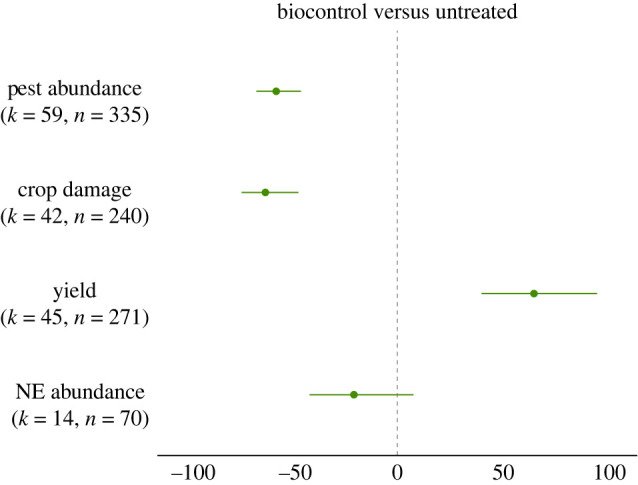
Changes in pest abundance, crop damage, yield and natural enemy (NE) abundance when biocontrol interventions are implemented compared to untreated crops (untreated/monocropping). The values are expressed in percentage with 95% bias-corrected confidence intervals. Results that cross zero indicate no significant difference between control and treatment groups. *k* = number of articles, *n* = number of effect sizes. (Online version in colour.)

Overall, relative to farms without any pest control method, biocontrol interventions had a strong negative effect on PA and CD, which were reduced by 55 and 60%, respectively (table 2; figure 2). Crops subject to biocontrol exhibited a 62% increase in Y. However, we found no significant overall effect of biocontrol on NEA (−19%) (figure 2). There was substantial heterogeneity for all outcome measures, suggesting unexplained variation (PA, *I^2^* = 54.98%; CD*, I^2^* *=* 51.35; Y*, I^2^* *=* 69.20%, NEA*, I^2^* *=* 92.35) (figure 2). Hence, we used meta-regression to elucidate the effect of potential moderators.

**Table 2.** Summary table of hierarchical meta-analysis models showing total heterogeneity, i.e. the effects of biocontrol interventions on the outcome measures without moderators (all), and heterogeneities explained by moderators: biocontrol intervention technique (botanical pesticides, field margins, intercropping and push–pull); crop type (cereal, fruits, fibre, pulses and vegetables); target pest taxon (Coleoptera, Hemiptera, Lepidoptera and Blattodea); and farming type (small farms and research farms) with the respective residual heterogeneities.

	**d.f.**	*Q*	*p*-value
**pest abundance**			
all	326	209370.95	<0.0001
biocontrol intervention technique	4	5.63	0.2133
*residuals*	322	205390.18	<0.0001
crop type	5	2.08	0.8368
* residuals*	321	58546.03	<0.0001
target pest taxon	5	3.61	0.6065
*residuals*	321	65549.49	<0.0001
farming type	1	2.74	0.0976
*residuals*	325	145118.45	<0.0001
**crop damage**			
all	239	13539.39	0.0120
biocontrol intervention technique	4	4.87	0.3003
*residuals*	235	11354.65	<0.0001
crop type	5	46.14	<0.0001
*residuals*	234	10586.19	<0.0001
target pest taxon	4	5.49	0.2402
*residuals*	235	11998.69	<0.0001
farming type	1	2.82	0.0931
*residuals*	238	13232.17	<0.0001
**yield**			
all	269	8706587.83	<0.0001
biocontrol intervention technique	4	23.13	<0.0001
*residuals*	265	8686621.24	<0.0001
crop type	5	1.26	0.9387
*residuals*	264	8697271.27	<0.0001
target pest taxon	5	3.77	0.5823
*residuals*	264	8691922.59	<0.0001
farming type	1	0.0679	0.7945
*residuals*	268	8706137.58	<0.0001
**natural enemy abundance**			
all	69	711.5758	<0.0001
biocontrol intervention technique	3	6.33	0.0966
*residuals*	66	626.78	<0.0001
crop type	4	8.94	0.0624
*residuals*	65	297.49	<0.0001
target pest taxon	2	12.61	0.0018
* residuals*	67	210.88	<0.0001
farming type	1	0.84	0.3580
*residuals*	68	303.21	<0.0001

### (b) Factors affecting biocontrol effectiveness

#### (i) Biocontrol intervention technique

**Figure 3.**
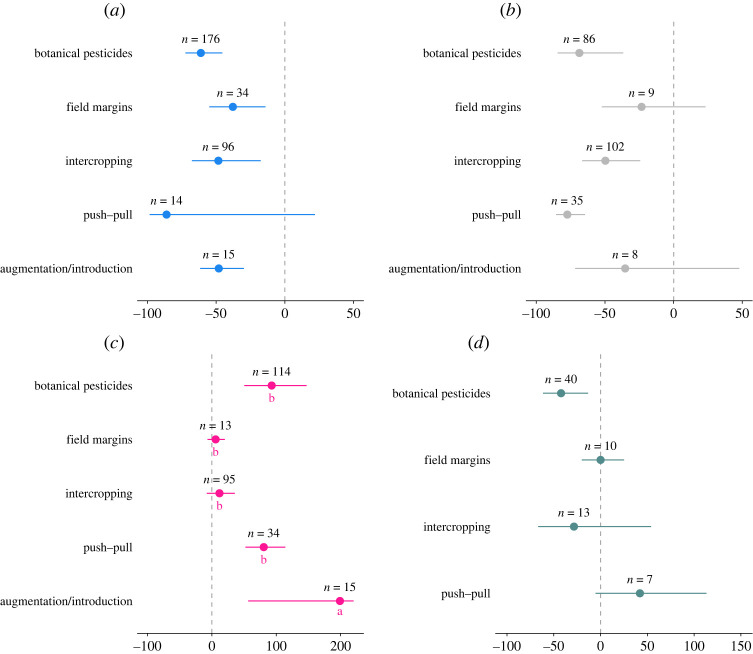
Changes in (*a*) pest abundance, (*b*) crop damage, (*c*) yield and (*d*) natural enemy abundance when biocontrol interventions are implemented compared to untreated crops (untreated/monocropping). The values are expressed in percentage with 95% bias-corrected confidence intervals categorized as botanical pesticides, field margins, intercropping, push–pull, and augumentation/introduction. Results that cross zero indicate no significant difference between control and treatment groups; *n* = number of effect sizes. (Online version in colour.)

Overall, the most tested biocontrol approaches were botanical pesticides (*n* = 244), followed by intercropping (*n* = 163) and push–pull (*n* = 46), followed by both field margins (*n* = 38) and augmentation/introduction (*n* = 38). We found that Y was significantly affected by the nature of the biocontrol intervention, with botanical pesticides and push–pull increasing Y by 92 and 80%, respectively (table 2; figure 3*c*). By contrast, the specific biocontrol technique adopted had no significant effect on PA, CD or contrasting effects on NEA.

#### (ii) Crop type

**Figure 4.**
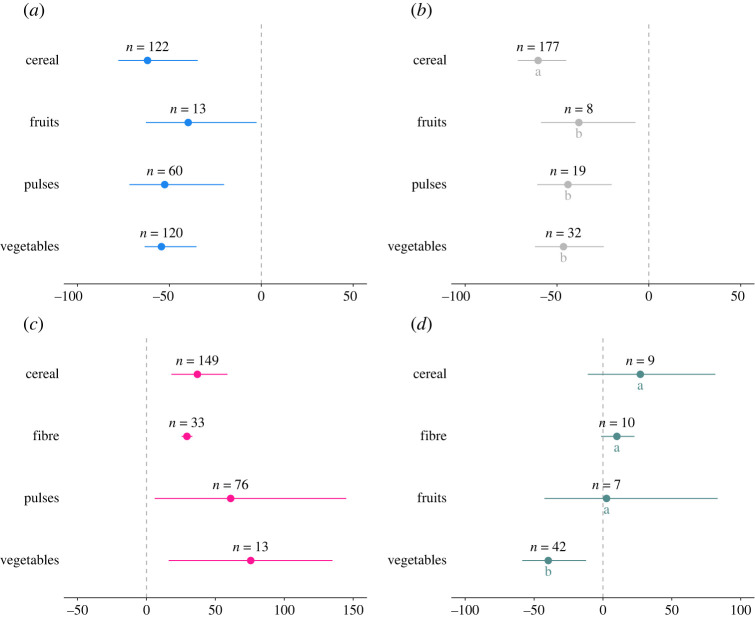
Changes in (*a*) pest abundance, (*b*) crop damage, (*c*) yield and (*d*) natural enemy abundance when biocontrol interventions are implemented compared to untreated crops (untreated/monocropping). The values are expressed in percentage with 95% bias-corrected confidence intervals categorized as cereal, fibre, fruits, pulses and vegetables where available. Results that cross zero indicate no significant difference between control and treatment groups; *n* = number of effect sizes. (Online version in colour.)

Across all outcome measures, the impact of biocontrol was measured predominantly in cereal crops (*n* = 457), followed by pulses (*n* = 155), vegetables (*n* = 207), fruits (*n* = 28) and fibres (*n* = 43). Biocontrol had an overall significant negative effect on PA across all crop types, with cereal pests showing a 61% reduction, followed by vegetable pests with a 54% reduction (table 2; figure 4*a*). PA in pulses and fruits showed a 52 and 39% decrease in pests, respectively (figure 4*a*).

We found that biocontrol had a strong negative effect on CD in all crop types tested: cereal: 60%, vegetables: 46%, pulses: 44% and fruits: 38% (figure 4*b*). Y was positively affected by biocontrol, but this varied according to crop type; Y in vegetables increased by 57% and pulses by 61% while cereals and fibres showed an increase of 36 and 29%, respectively (figure 4*c*). The specific crop type in which biocontrol interventions were tested did not influence the abundance of natural enemies (NEA, *p* = 0.06; figure 4*d*).

#### (iii) Target pest taxon

**Figure 5.**
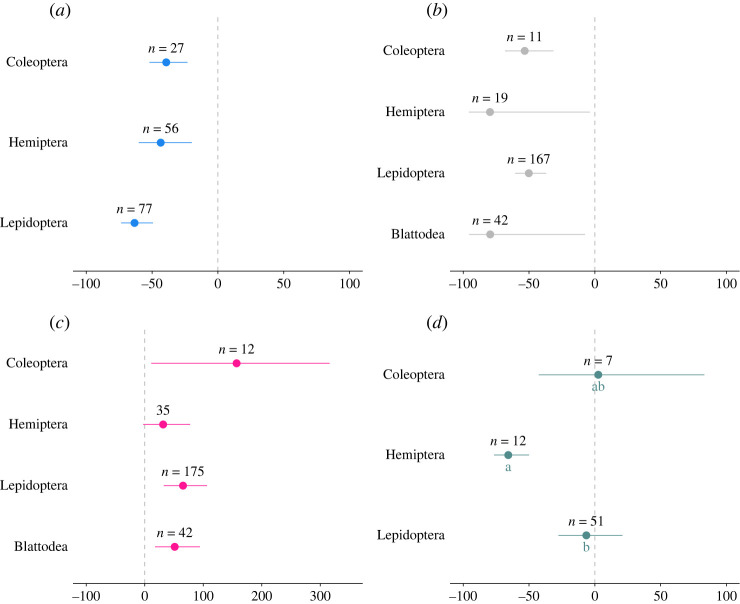
Changes in (*a*) pest abundance, (*b*) crop damage, (*c*) yield and (*d*) natural enemy abundance when biocontrol interventions are implemented compared to untreated crops (untreated/monocropping). The values are expressed in percentage with 95% bias-corrected confidence intervals categorized as Coleoptera, Hemiptera, Lepidoptera and Blattodea where available. Results that cross zero indicate no significant difference between control and treatment groups; *n* = number of effect sizes. (Online version in colour.)

Biocontrol interventions had a significant negative effect on the abundance of all pest taxa, with lepidopteran pests showing the greatest decline (−63%) (table 2; figure 5*a*). The CD of all taxa was strongly negatively affected by biocontrol interventions, with damage caused by Blattodea showing a 79% reduction with biocontrol implementation (figure 5*b*). We found that exposure to biocontrol interventions had a significant positive effect on Y where Coleoptera, Lepidoptera and Blattodea were the targeted pests (figure 5*c*, Coleoptera: 157%; Lepidoptera: 65%; and Blattodea 51%). There was no detectable effect of pest taxon on NEA response to biocontrol (figure 5*d*).

#### (iv) Comparison of research and farmers' fields

**Figure 6.**
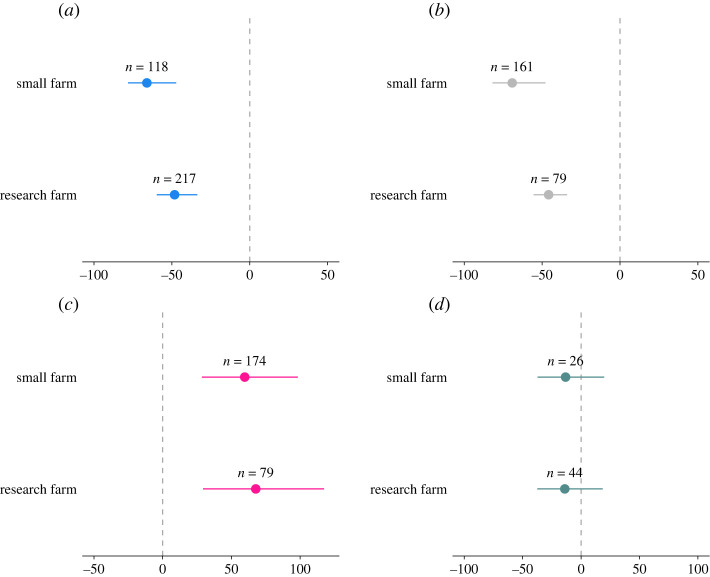
Changes in (*a*) pest abundance, (*b*) crop damage, (*c*) yield and (*d*) natural enemy abundance when biocontrol interventions are implemented compared to untreated crops (untreated/monocropping). The values are expressed in percentage with 95% bias-corrected confidence intervals categorized as small farms and research farms. Results that cross zero indicate no significant difference between control and treatment groups; *n* = number of effect sizes. (Online version in colour.)

Across all outcome measures, effect sizes did not differ significantly between farming types. In terms of cropping systems, the size of the negative effect of biocontrol on PA was marginally higher in smallholder farms (66%) than in research farms (48%) (table 2; figure 6*a*). CD showed a similar pattern, where reduction in small holder farms (−69%) marginally exceeded that of research farms (45%) (figure 6*b*). With regards to Y, the proportional increase was almost equal in the two cropping types (small farm: 59% and research farm 67%). In neither case was NEA affected by biocontrol interventions.

#### (v) Comparison with synthetic pesticides

The effectiveness of biocontrol interventions compared to synthetic pesticides was measured mostly for botanical pesticides (*n* = 339), followed by intercropping (*n* = 26) and augmentation/introduction (*n* = 23). We found no studies comparing the effect of field margins or push–pull with pesticides on their ability to control crop pests.

**Figure 7.**
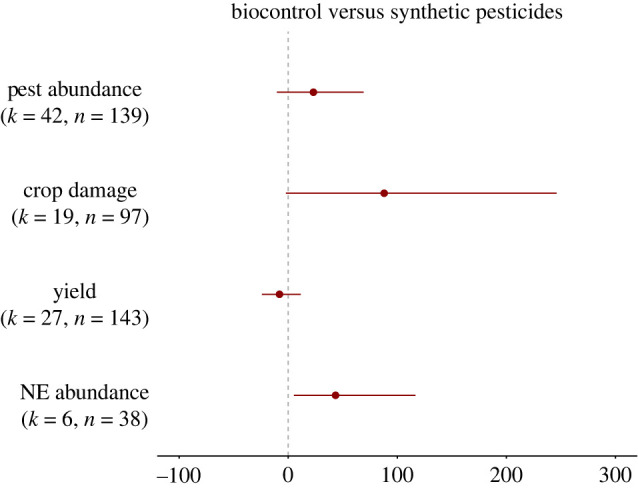
Changes in pest abundance, crop damage, yield and natural enemy (NE) abundance when biocontrol interventions are implemented compared to crops treated with synthetic pesticides. The values are expressed in percentage with 95% bias-corrected confidence intervals. Results that cross zero indicate no significant difference between control and treatment groups. *k* = number of articles, *n* = number of effect sizes. (Online version in colour.)

Although biocontrol interventions showed marginally greater PA and damage, and reduced Y compared to synthetic pesticides, we found no significant difference between the two treatments (figure 7; PA: 23%; CD: 87%; Y: −7%; NEA: 43%). Conversely, the abundance of natural enemies was significantly greater following biocontrol implementation compared to the application of synthetic pesticides (43%) (figure 7).

### (c) Landscape configuration

Our search yielded seven studies that explored the effect of landscape configuration on biocontrol delivered to crops in SSA. Four studies showed a positive effect of proximity to natural habitat, or proportion of natural habitat within a given buffer, on natural enemy activity (i.e. parasitism and predation) [45–48]. Only three studies explored the interactive effects of landscape complexity and farm management on pest control effectiveness [49–51]. All studies found an interactive effect of management and landscape configuration, though the low sample size did not allow for quantitative analysis here.

## 4. Discussion

In this study, we identified the overall effectiveness of biocontrol techniques in controlling insect pests of crops in SSA, and identified patterns across biocontrol interventions, pest taxa, crop types and experimental design. Using a set of hierarchical meta-analyses, we found that biocontrol interventions effectively reduced PA and CD by over 50%, while increasing Y by more than 60%. The size of the yield increases highlights the great challenge posed by insect pests to smallholder crop production, which is in line with recent evidence estimating high crop losses to pests, especially in the absence of any control intervention [52,53]. The substantial yield increase that biocontrol can provide could have an enormous impact on sub-Saharan food security if these practices are scaled up to regional level. Crucially, we showed comparable performance of biocontrol and synthetic pesticides on PA, CD and Y, and a significant reduction in the loss of natural enemies, particularly following botanical pesticides application.

### (a) Biocontrol effectiveness across biocontrol intervention techniques

PA and CD were negatively affected by biocontrol across all interventions. Push–pull and botanical pesticides had the greatest effect on Y, increasing production by 92 and 80%, respectively. This may be owing to the highly effective companion crops used in push–pull technologies, which release bioactive chemicals that repel pests and attract natural enemies, while also suppressing *Striga*, a parasitic weed which causes up to 100% yield losses across SSA [54]. The large yield increase observed in our synthesis may be owing to a combination of the pest repellent and weed suppression abilities of push–pull implementation. Our findings reveal the potential of botanical pesticides to be an effective method of pest control in SSA. However, two-thirds of the studies included here were carried out on research farms, which may be under more controlled settings compared to more realistic field conditions, potentially inflating the observed effect size.

Our review captured a small number of studies on classical biocontrol interventions, including augmentation, despite successful examples, such as the control of the cassava mealybug (*Phenacoccus manihoti*) by the encyrtid wasp (*Anagyrus lopezi*) [55]. Conceivably these interventions may be hampered by the high costs involved in their research and production, such as insect rearing facilities [56], and the growing concerns on the environmental risks of releasing exotic species [57]. Therefore, they may only be implemented for highly widespread and devastating pests such as the cassava mealybug or the tomato leaf miner (*Tuta absoluta*).

### (b) Biocontrol effectiveness across crop type and pest taxon

Cereals were the most studied crops in our meta-analysis, conceivably because they play a central role in the region's food security, accounting for about 50% of total crop area and caloric intake [58]. Nonetheless, other crop types, such as fruits, pulses and fibre should be included in future research in this area. Our study provides strong evidence of the effectiveness of biocontrol across all taxa, particularly against lepidopteran crop pests. The potential of biocontrol to reduce cereal CD by 60% is encouraging given the devastating damage caused, particularly on maize, by caterpillars including fall armyworm (*Spodoptera frugiperda*), diamondback moth (*Plutella xylostella*), crambid cereal stemborer (*Chilo partellus*) and maize stemborer (*Busseola fusca*).

### (c) Biocontrol effect on natural enemies and non-target pests

Understanding the effect of biocontrol on natural enemy populations is crucial as they are both an indication of pest control potential and a measure of the impact of the pest control method on non-target species. Our results showed no overall change in NEA following biocontrol application when compared to untreated fields, although we found a significant decline in NEA following botanical pesticide application. The most likely explanation for this is that the interventions have reduced prey availability for natural enemies, making them move to other more profitable foraging locations, which has been shown in previous studies on intercropping where pest number, not the interventions, influenced PA [59,60]. However, the direct negative impact of some interventions, such as some broad-spectrum botanical pesticides, cannot be excluded [61]. The existing evidence for the effect of botanical pesticides on non-target species is conflicting, with some research showing that plant extracts such as neem, garlic and eucalyptus may cause mortality and have sub-lethal effects on beneficial insects [62,63], while other studies found no detrimental effect of pepper and garlic extract on natural enemy populations [24,64]. More research is needed to draw robust inferences on the repercussions of botanical pesticides on beneficial/non-target species before considering large-scale adoption.

Evidence is more consistent on the positive response of natural enemy populations to biocontrol interventions such as push–pull and field margins [65,66], which is in line with evidence from the global north on the benefits of habitat enhancement on natural enemy density and diversity [67,68]. However, we found that only 14% of the studies measured NEA following biocontrol application in SSA. NEA should be measured more consistently in future studies to further elucidate direct and indirect effects of biocontrol on non-target species.

Furthermore, the most common outcome measures reported in the studies focussed on the abundance of pests and/or natural enemies, while we did not find studies measuring their species diversity or functional group diversity. However, it has been shown that biocontrol is strengthened by increased natural enemy richness [69,70], and this is consistent across temperate and tropical regions [71]. Ecosystem functioning can be stabilized by functional redundancy, by enabling functional groups to compensate for individual species fluctuations and increase the resilience of ecosystem against species loss [72,73]. This is particularly relevant to understand the long-term impact of biocontrol on natural enemy communities and their pest suppression ability and should be explored in future research.

### (d) Biocontrol effectiveness compared to synthetic pesticides

When compared to synthetic pesticides, biocontrol interventions had a similar impact on PA and CD, which is a critical finding for farmers who cannot access or afford chemicals. Crucially, NEA was significantly reduced after synthetic pesticide application even over the short timescales of the studies examined. In the long term there could be greater reductions in pest and crop damage following biocontrol as a result of more abundant and diverse communities of natural enemies. In terms of a reduction in the negative environmental impacts associated with chemical pesticides, the benefits provided by more resilient natural enemy populations could be one of several indirect positive effects of opting out of conventional pesticide use. It is worth noting that most comparisons with synthetic pesticides were measured against botanical pesticides, therefore inferences for other biocontrol methods should be made with caution. Future research should aim to determine the effectiveness of biocontrol approaches, such as push–pull, when compared to synthetic pesticides to fill this knowledge gap.

A possible limitation of this study is the potential selection bias towards significant results, causing an over-representation in the published literature, a criticism that could be levelled against all meta-analyses. The two tests we used to assess publication bias yielded conflicting results; hence, it is hard to know with certainty the scale of publication bias towards results where an effect was found. However, we show that crop losses to pests are significantly higher in untreated fields, supporting the idea that any crop protection intervention has the potential to improve yields substantially. The size of the yield gains shown in the current meta-analysis suggests there is a big opportunity to raise yields with biocontrol interventions.

### (e) Landscape configuration and biocontrol

Our study set out to answer the question, ‘does the surrounding landscape configuration affect the effectiveness of biocontrol interventions?’, which has led to positive responses of natural enemies to landscape complexity in studies outside of the SSA region [13]. However, we found a paucity of studies investigating either the effect of landscape configuration on biocontrol effectiveness, or the relationship between landscape configuration and NEA. The research we found indicated a significant decrease of natural enemy density and predation/parasitism activity with isolation from natural habitat (e.g. [45,48]). This is in line with recent research showing a similar effect of landscape complexity on pollinators and natural enemies in sub-Saharan regions [74,75] and a larger body of research particularly in the global north [13,19,76].

Furthermore, the sparse evidence we found focusing on the effect of landscape configuration on biocontrol effectiveness showed inconsistent results. Midega *et al.* [49] found that semi-natural habitat acted as a source of lepidopteran pests to the maize crop fields in Kenya, while Kebede *et al*. [50] demonstrated that landscape simplification overrode the effect of intercropping practices and was the main driver of pest infestation levels. A key avenue for future research would involve large scale studies to identify clear patterns in the relationship between landscape complexity and natural enemy activity and the ecosystem service delivered to sub-Saharan agricultural systems. Additionally, recent evidence from SSA showed that natural enemy diversity in crop fields is dependent on the land management of neighbouring fields [29]. This highlights the need for further multi-scale studies to identify potential variation in biocontrol effectiveness across different land management contexts.

## 5. Conclusion

Our findings provide, to our knowledge, the first quantitative synthesis of biocontrol effectiveness in SSA, indicating that biocontrol interventions have the potential to substantially reduce CD, increase Y while maintaining natural enemy populations within sub-Saharan agricultural systems. Our results further suggest that biocontrol has comparable performances to synthetic pesticides with reduced adverse impact on beneficial insects and ecosystems, which makes it an effective alternative intervention for farmers who do not have access to pesticides, while it can maintain Y without associated negative pesticide effects. Given the case against chemical use in Africa [9], the efficacy of biocontrol options demonstrated in this meta-analysis provides a strong regionally focused evidence base for policy- and decision-makers to be persuaded of their validity as an alternative to chemicals. Overall, our results encourage an update to national agricultural policies, which inconsistently feature biocontrol, and can support policy makers in the design of more resilient and sustainable pest management practices across the sub-Saharan region.

## Data Availability

Data used in these analyses are available from the Dryad Digital Repository: https://doi.org/10/50161/dryad.s1rn8pkbw [77]. The data are provided in the electronic supplementary material [78].
